# Divergent behaviors and underlying mechanisms of cell migration and invasion in non-metastatic T24 and its metastatic derivative T24T bladder cancer cell lines

**DOI:** 10.18632/oncotarget.2680

**Published:** 2014-11-04

**Authors:** Honglei Jin, Yonghui Yu, Young Hu, Chris Lu, Jingxia Li, Jiayan Gu, Liping Zhang, Haishan Huang, Dongyun Zhang, Xue-Ru Wu, Jimin Gao, Chuanshu Huang

**Affiliations:** ^1^ Zhejiang Provincial Key Laboratory for Technology & Application of Model Organisms, School of Life Sciences, Wenzhou Medical University, Wenzhou, Zhejiang, China; ^2^ Nelson Institute of Environmental Medicine, New York University School of Medicine, Tuxedo, NY, USA; ^3^ Departments of Urology and Pathology, New York University School of Medicine, New York, NY, USA; ^4^ Veterans Affairs New York Harbor Healthcare System Manhattan Campus, New York, NY, USA

**Keywords:** bladder cancer, migration, invasion, SOD2, MMP-2

## Abstract

Previous studies on cancer cell invasion were primarily focused on its migration because these two events were often considered biologically equivalent. Here we found that T24T cells exhibited higher invasion but lower migration abilities than T24 cells. Expression of Rho-GDPases was much lower and expression of SOD2 was much higher in T24T cells than those in T24 cells. Indeed, knockdown of SOD2 in T24T cells can reverse the cell migration but without affecting cell invasion. We also found that SOD2 inhibited the JNK/c-Jun cascade, and the inhibition of c-Jun activation by ectopic expression of TAM67 impaired Rho-GDPases expression and cell migration in T24T shSOD2 cells. Further, we found that Sp1 can upregulate SOD2 transcription in T24T cells. Importantly, matrix metalloproteinase-2 (MMP-2) was overexpressed in T24T and participated in increasing its invasion, and MMP-2 overexpression was mediated by increasing nuclear transport of nucleolin, which enhanced mmp-2 mRNA stability. Taken together, our study unravels an inverse relationship between cell migration and invasion in human bladder cancer T24T cells and suggests a novel mechanism underlying the divergent roles of SOD2 and MMP-2 in regulating metastatic behaviors of human bladder T24T in cell migration and invasion.

## INTRODUCTION

The acquisition of migratory and invasive properties by cancer cells is a key development preceding metastasis [1, 2]. Previous studies often consider migration and invasion are biologically equivalent, but our result shows that they are two different events [3-5]. Cell migration comprises a number of individual steps: loss of cell-cell adhesion, membrane protrusion in the direction of movement, de-adhesion from the extracellular matrix in the rear, re-adhesion in front, and contraction of the actin cytoskeleton to drive the cell body forward. Rho-GTPases, including CDC42 and Rac1, play essential roles in the process of cell migration, including adhesion, subsequent loss of attachment, re-adhesion, polarization, and cytoskeletal organization [6]. Given the importance of CDC42 and Rac1 in the cell migratory events, perturbation of the natural balance of these GTPases in a cell may ultimately lead to phenotypes of metastasis. Cancer cell metastasis also consists of multiple steps: infiltration into the linking tissue, entrance into the blood or immune system, and the formation of a new tumor in a distant organ. Hydration of the extracellular matrix is crucially important for cancer cell invasion and metastasis, a process that tumor cells promote through the secretion of proteins such as matrix metalloproteinase-2 (MMP-2) and MMP-9. MMP-2, also known as the 72kDa type IV collagenase, is an enzyme that degrades type IV collagen, a major structural component of the basement membrane. MMP-2 has been associated with the metastasis of many cancers, including colorectal cancer [7], ovarian cancer [8], and breast cancer [9], but it does not have a clear association with bladder cancer invasion.

Superoxide dismutases (SOD) are a class of enzymes that catalyze the dismutation of superoxide into oxygen and hydrogen peroxide. There are three forms of superoxide dismutases: cytoplasmic SOD (SOD1), mitochondrial SOD (SOD2), and extracellular SOD (SOD3) [10]. SOD2 transforms toxic superoxide, a by-product of the mitochondrial electron transport chain, into hydrogen peroxide and diatomic oxygen, and involves the process of a reactive oxygen species [11]. Recent studies have shown that SOD2 is consistently increased in high-grade and advanced-stage bladder cancer and plays an important role in cancer development and cancer metastasis [12, 13]. The transcription factor activator protein-1 (AP-1) regulates a wide range of cellular processes, including cell proliferation, survival, and differentiation [14]. C-Jun is a member of AP-1, and has been proposed to play an important role in carcinogenesis and cancer development. C-Jun activity can be regulated by several mechanisms, including the activation of the c-Jun N-terminal kinase (JNK) [15, 16]. The activation of JNK leads to the phosphorylation and activation of a number of signal pathways associated with cancer development and metastasis [17]. SOD2, CDC42/Rac1, and JNK/c-Jun are all known to be involved in cancer development and migration [18-21]. However, the roles of SOD2, CDC42/Rac1, and JNK/c-Jun in bladder cancer aggression and migration remain largely unexplored.

To gain insights into the biological significance of these molecules in the context of bladder cancer cell migration and invasion, we chose to comparatively study the migratory and invasive properties and their underlying mechanisms of a pair of isogenic bladder cancer cell lines, T24 and T24T, with distinct metastatic characteristics. While T24 cells have limited metastatic potential, its derivative T24T cells have highly metastatic. Protein and mRNA expression of the above molecules in T24 and T24T cells were examined and potential relationships among the proteins were investigated. These studies provide new insights into the molecular basis of bladder cancer cell migration and invasion, and reveal novel therapeutic targets for the cell metastasis of bladder cancers.

## RESULTS

### Inverse relation between cell migration and invasiveness in highly metastatic human bladder cancer T24T cells and its parental T24 cells

**Figure 1:**
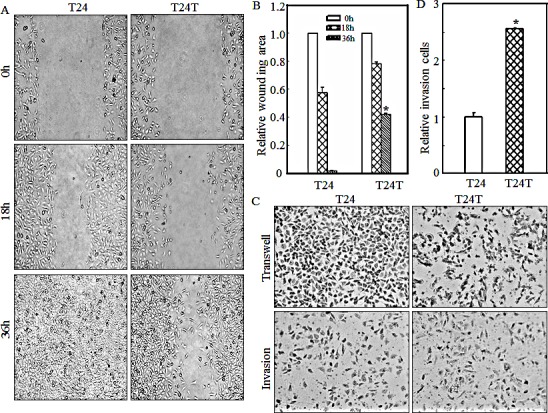
T24T has increased cell invasiveness with decreased cell migration abilities compared to its parental T24 cells (A) Bladder cancer cells T24 and T24T were seeded to 6-well plates. When cell confluence reached 60~70%, the wounds were made using sterile tips. The images were taken under the inverted microscope at the indicated times. (B) The wound area was quantified using cell migration analysis software and the results were presented as indicated. (C) Invasion abilities of T24 and T24T cells were determined using BD BioCoat^TM^Matrigel^TM^ Invasion Chamber. The migration ability was determined by using the empty insert membrane without the matrigel. The invasion ability was assessed by using the same system except that the matrigel was applied. (D) The invasion rate was normalized with the insert control according to the manufacturer's instruction. The asterisk (*) indicates a significant difference between T24 and T24T cells (p< 0.05). The values shown are mean ± SD from three independent experiments.

The T24/T24T system is a well-established bladder cancer model that involves T24 cells, with limited cancer metastasis, and T24T cells, derived from T24 cells that possess significant cancer metastasis. The salient difference between these two cell lines provides an ideal model to investigate the molecular basis of bladder cancer metastasis. To investigate the cell migration of T24 and T24T cells, the wound healing assay was performed as described in “Materials and Methods” and as shown in Figs.1A and 1B. Thirty-six hours after wounding, the T24 line had complete healing, while T24T exhibited only partial healing, indicating that T24T cells had a lower migration abilities as compared with T24 cells. The results obtained from a transwell assay showed that T24T cells also decreased its migration abilities (Fig. 1C, upper panel), while increased its invasive abilities in comparison to T24 cells (Figs. 1C and 1D). These experiments reveal the inconsistent nature of the relationship between a cell's invasive and migratory abilities in both T24 and T24T cells, suggesting that high cell migratory activity does not necessarily mean high invasive activity.

### SOD2 is involved in suppression of T24T cell migration

**Figure 2:**
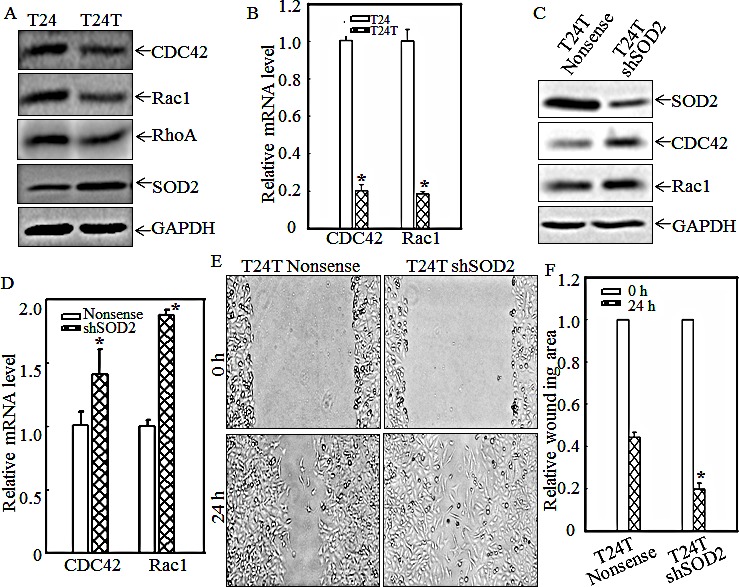
SOD2 inhibited CDC42 and Rac1 expression and thus, cell migration in human bladder T24T cells (A and C) the indicated cell extracts were subjected to Western blot for determination of expression of SOD2, Rac1, RhoA and CDC42. (B and D) Total RNA was extracted from T24, T24T and T24T nonsense as well as T24T shSOD2 cells. After reverse transcription, the mRNA levels of Cdc42 and Rac1 in T24, T24T cells and their transfectants, were evaluated by using real time PCR assay. (E and F) T24T transfectants were each seeded to 6-well plates as indicated. When cell confluence reached 60~70%, the wounds were made using sterile tips. And the images were taken by under the inverted microscope at 24 hrs after wounding (E). The wound area was quantified using cell migration analysis software and the results were presented as indicated (F).

CDC42, Rac1, and RhoA are all members of the Rho family of small GTPases, and are key regulators of actin polymerization and therefore cell migration [22-24]. To investigate the molecular basis of the inconsistent relationship between cell migration and invasion, as observed in T24 and T24T cells, the expression levels of the small GTPases, CDC42, Rac1 and RhoA, were determined in both cell lines. As shown in Fig. 2A, CDC42, Rac1 and RhoA protein expressions were dramatically decreased in T24T cells in comparison with those of T24 cells (Fig.2A). The mRNA levels of cdc42 and rac1 were also analyzed using real-time PCR. Consistent with their protein levels in T24 and T24T cells, both mRNA levels were remarkably decreased in T24T cells compared to those in T24 cells (Fig.2B). These results revealed that CDC42/Rac1 might participate in cell migration inhibition in T24T cells. Previous studies have shown that SOD2, a well-known antioxidant enzyme, could play a part in the promotion or progression of certain cancers. Some reports show that SOD2 can induce the migration and invasion of tongue squamous cell carcinomas [19], while others have shown that SOD2 is lower in cancer cells [25] and that the reduced SOD2 expression might be related to malignant cancer progression [26]. SOD2 and SOD3 could also suppress vascular smooth muscle cell and inflammatory cell migration, respectively [27, 28]. We, therefore, evaluated the SOD2 expression of T24 and T24T cells. The results indicated that SOD2 expression was significantly increased in T24T cells (Fig. 2A), which suggests that this higher expression of SOD2 might mediate restraining cell migration in T24T cells. To further reveal the function of SOD2 in T24/T24T cell migration, small hairpin RNA specifically targeting SOD2 (shSOD2) was transfected into T24T cells, as identified in Fig. 2C. The knockdown of SOD2 in T24T cells increased the protein and mRNA levels of both CDC42 and Rac1 (Figs.2C and 2D) and also enhanced cell migratory activity compared with that of a T24T nonsense cell (Figs. 2E and 2F). Therefore, we concluded that SOD2 overexpression in T24T cells inhibits CDC42 and Rac1 expression, which subsequently results in the suppression of T24T cell migration.

### SOD2 inhibits T24T cell migration by targeting JNK/AP-1/GTPase cascade

**Figure 3:**
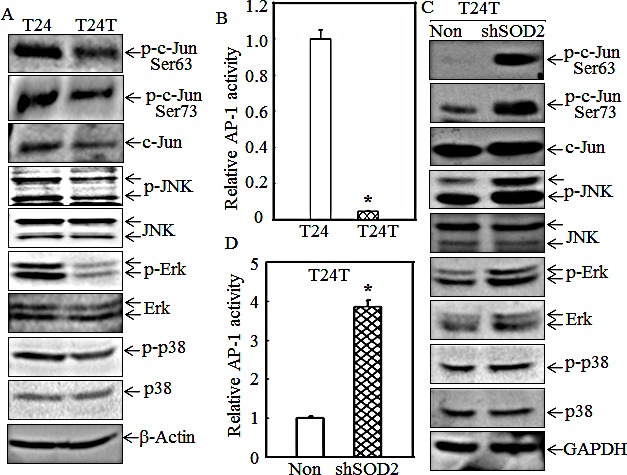
SOD2 inhibited JNK-c-Jun/AP-1 activation in T24T cells (A and C) The cell extracts from T24, T24T cells and T24T transfectants were subjected to Western blot for determination of protein expression as indicated. (B and D) T24 and T24T cells or T24T/nonsense and T24T/shSOD2 cells were co-transfected with AP-1 luciferase reporter and pRL-TK. 24 hrs after transfection, the cells were extracted and subjected to determination of the luciferase activities. The results were presented as AP-1 activity relative to T24 cells or T24T nonsense control with normalized by internal TK activity. The asterisk (*) indicated a significant difference between the paired cells as indicated (p< 0.05). The bars were shown as mean ± SD from three independent experiments.

The MAPKs pathway is critical to cancer cell motility [29, 30]. To evaluate the potential effect of MAPKs and their relationship with SOD2 in regulation of T24/T24T cell migration, the JNK, Erk and p38 activation levels were evaluated and compared among T24, T24T and T24T shSOD2 cells. The phosphorylation levels of JNK and Erk were remarkably decreased while p38 was only slightly reduced in T24T cells as compared with T24 cells; however the expression of total JNK, p38 and Erk protein were all comparable in T24 and T24T cells (Fig. 3A). The activation of their downstream transcription factor c-Jun, a key transcription factor AP-1 component, was also compared and its phosphorylation at Ser63 and Ser73 were significantly lowered in T24T cells (Fig.3A). Consistent with the phosphorylation levels, the AP-1 luciferase assay also demonstrated that its transactivation was also dramatically suppressed in T24T cells in comparison with that in T24 cells (Fig. 3B). Very interestingly, the T24T shSOD2 cells had markedly higher activation of JNK, Erk, and c-Jun, while the phosphorylation and total level of p38 was comparable between T24T shSOD2 and its vector control transfectant T24T nonsense cells (Figs. 3C and 3D). These results indicate that SOD2 is implicated in inhibiting the function of JNK, Erk and their downstream transcription factor, c-Jun/AP-1, and that p38 is probably not involved in SOD2's regulation of cell migration.

**Figure 4:**
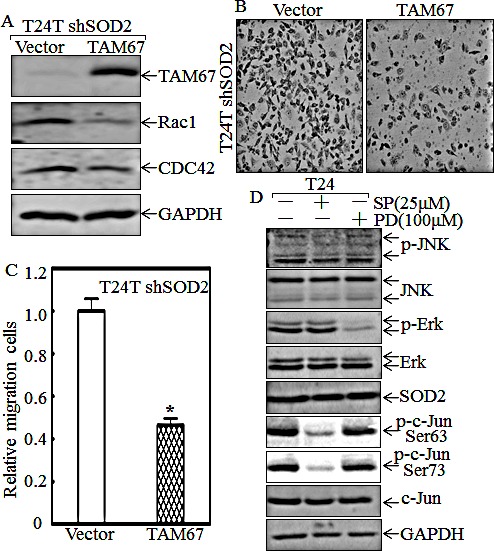
C-Jun mediates SOD2-regulated Rac1 and CDC42 expression and cell migration of T24T cells (A) The cell extracts from T24T shSOD2 (Vector) and T24T shSOD2 (TAM67) transfectants were subjected to Western blot for determination of protein expression as indicated. (B and C) Migration abilities of T24T shSOD2 (Vector) and T24T shSOD2 (TAM67) cells were determined using BD BioCoat^TM^ Chamber that the matrigel was not applied. The cells were stained with Giemsa, and images of the cells on the outside of the chamber were taken under microscope (B) and the cell number was counted by the software “Image J”. The asterisk (*) indicated a significant difference between indicated two transfectants (p< 0.05). The bars were shown as mean±SD from three independent experiments. (D) T24 cells were seeded into a 6-well plate, and when cell confluence reached 70~80%, the cells were pretreated with or without SP600125 (25μM), or PD98059 (100μM) for 12 hrs. The cell lysate was subjected to Western Blot assay for determination protein levels using specific antibodies as indicated.

To investigate the effect of c-Jun on CDC42/Rac1 expression and cell migration, dominant negative c-Jun mutant, TAM67, was stably transfected into T24T shSOD2 cells. The results showed that CDC42/Rac1 expression levels (Fig. 4A), along with the cell migratory abilities were dramatically decreased (Figs. 4B and 4C); strongly indicating that c-Jun is critical for CDC42/Rac1 expression and cell migration. To investigate the effects of JNK and Erk on c-Jun phosphorylation, the inhibitors SP600125 (specific to JNK) and PD98059 (specific to MEK/Erk) were employed in the T24 cell assay. The results of the inhibition assay are shown in Fig. 4D. SP600125 and PD98059 successfully attenuated the phosphorylated level of JNK and Erk, respectively, but the c-Jun phosphorylation at Ser63 and Ser73 were reduced only in the cells treated with JNK inhibitor SP600125 (Fig. 4D). Moreover, the expression of SOD2 was comparable among all group cells. This suggests that SOD2 is an upstream effect or of JNKs responsible for c-Jun activation, by which participate in SOD2 regulation of cell migration, whereas Erk is not involved in c-Jun-mediated cell migration in T24 cells.

### Transcription factor Sp1 overexpression mediates SOD2 transcription and protein expression in T24T cells

**Figure 5:**
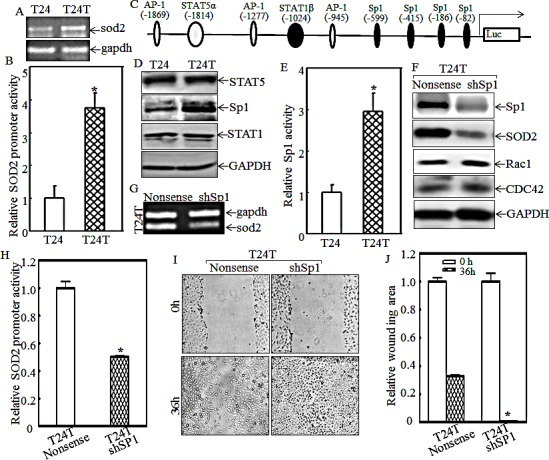
Sp1 overexpression and activation mediates SOD2 transcription and in turn inhibits Rac1 and CDC42 expression and cell migration in T24T cells (A) The mRNA levels of *sod2* in T24 and T24T cells were analyzed using RT-PCR. (B) SOD2 promoter-driven luciferase activity was evaluated in T24T cells. The results were normalized by internal TK activity. (C) The potential transcription factor binding sites of the SOD2 promoter. (D) The expression levels of Sp1, STAT1 and STAT5, were determinated by Western blot as indicated. (E) The Sp1-dependent transcriptional activity was evaluated by using Sp1-dependent luciferase reporter. (F) SOD2 and the downstream effectors were evaluated by using Western Blot after the knockdown of Sp1 in T24T cells. (G) sod2 mRNA levels were evaluated in T24T nonsense and T24T shSp1 transfectants. (H) SOD2 promoter transcription activity in T24T nonsense and T24T shSp1 transfectants were determinated by co-transfection of SOD2 promoter-driven luciferase reporter together with pRL-TK. The results were normalized by internal TK activity. (I and J) The wound healing assay was used to determine the migratory abilities of the T24T nonsense and T24T shSp1 transfectants, and the wound area was quantified using cell migration analysis software (J).

To evaluate the mechanisms underlying SOD2 overexpression in T24T cells, sod2 mRNA levels were first determined using RT-PCR assay. As shown in Fig. 5A, the sod2 mRNA level was higher in T24T than that in T24 cells. It is known that mRNA expression could be regulated at either transcription level or mRNA stability level. Following the line of transcription level, the SOD2 promoter-driven luciferase reporters were transfected into both T24 and T24T cells and SOD2 transcription expression was determined and compared between two cell lines as described in the “Materials and Methods”. The results showed that the SOD2 promoter activity of T24T cells was almost 4-fold higher than that of T24 cells (Fig.5B), suggesting that the increased SOD2 expression in T24T cells occurred at the transcriptional level. Therefore, the potential transcriptional factors that can bind to the SOD2 promoter region, including AP-1, STAT, and multiple Sp1s, were analyzed as shown in Fig. 5C, and Sp-1-dependent transcription activities in T24 and T24T cells were evaluated by stably transfection of Sp-1-dependent luciferase reporters into T24 and T24T cells, respectively. The result demonstrated that both Sp1 expression and Sp1-dependent transcription activity were significantly increased in T24T cells compared to T24 cells (Figs. 5D and 5E). To determine the role of Sp-1 in SOD2 expression in T24T cells, Sp1-specific shRNA was used to knockdown of Sp1 expression in T24T cells (Fig. 5F). The protein and mRNA level of SOD2 expression was remarkably decreased in T24T knockdown Sp1 (T24T shSp1) cells (Fig. 5F and 5G). Consistently, the SOD2 promoter-driven luciferase activity was also depressed in T24T shSp1 cells (Fig. 5H). Importantly, the cell migration activity of T24T shSP1 cells were profoundly increased compared with that of T24T nonsense cells (Figs. 5I & 5J). These suggest that transcriptional factor Sp1 overexpression and activation is responsible for promoting SOD2 expression, which subsequently decreases T24T cell migration abilities.

### Increased MMP-2 expression contributes to T24T invasion

**Figure 6:**
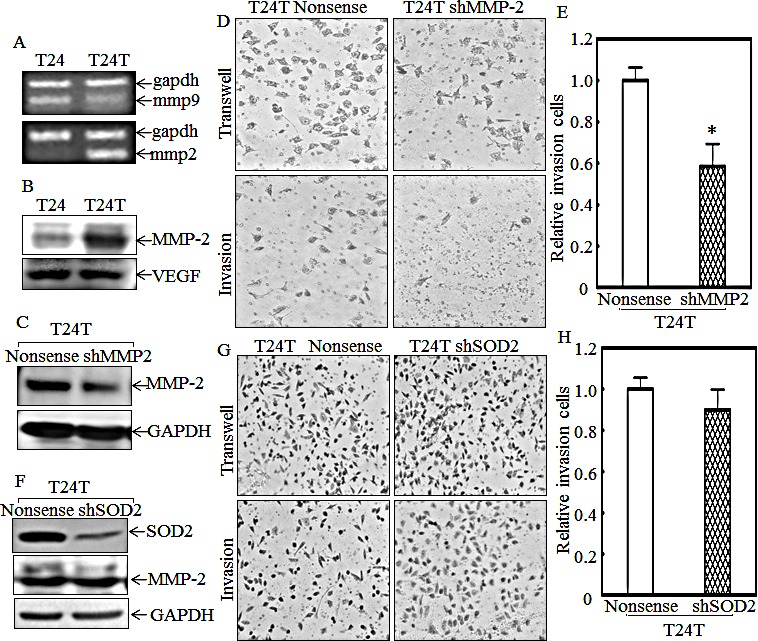
MMP-2, but not SOD2, contributes to high invasion of T24T cells (A) mRNA expression of mmp-2 and mmp-9 was evaluated by RT-PCR in T24 and T24T cells. (B) MMP-2 and VEGF protein were determinated by Western Blot. (C-E) Stable transfectants, T24T/nonsense and T24T/shMMP-2, were identified by Western Blot (C) and their invasive abilities were evaluated by Transwell invasion assay (D). The migration ability was determined using the empty insert membrane without the matrigel, while the invasion ability was evaluated by using the same system except that the matrigel was applied. (E) The invasion rate was normalized to the insert control according to the manufacturer's instruction and the results were presented as the invasion cells relative to T24T nonsense control transfectant. (F) The cell extracts from T24T shSOD2 and its nonsense control transfectants were subjected to Western Blot for determination of its effect on MMP-2 expression. (G and H) T24T shSOD2 and its nonsense control transfectant were used for determination of their invasion abilities in transwell invasion assay (G). The migration ability was determined by using the empty insert membrane without the matrigel, while the invasion ability was evaluated by using the same system except that the matrigel was applied. The invasion rate was normalized with the insert control according to the manufacturer's instruction and the results were presented as the invasion cells relative to T24T nonsense control transfectant (H).

Matrix metalloproteinase 2 (MMP-2) and MMP-9 are reported to enhance cancer cell invasion via degradation of type IV collagen [8, 31]. To explore whether MMP-2 and MMP-9 are involved in regulation of human bladder cancer cell invasion, we first compared their expression levels between T24 and T24T cells. The results showed that MMP-2 was increased in both protein and mRNA level in T24T, but MMP-9 was actually decreased in mRNA level (Figs. 6A and 6B). Another protein, VEGF, was also reported to promote cancer cell invasion and metastasis [32], and was also measured in T24 and T24T cells. However, its expression had no significant difference between the two cell lines (Fig. 6B). So we anticipated that the MMP-2 overexpression in T24T cells might be a key factor for its increased invasion and metastasis. To further test this notion, we transfected MMP-2 specific shRNA into T24T cells, and the stable T24T shMMP-2 cells were established as demonstrated with MMP-2 protein knockdown level (Fig. 6C). The knockdown of MMP-2 decreased the T24T cell invasion abilities in comparison to its nonsense transfectants (Figs. 6D and 6E). These results indicate that MMP-2 is a critical factor in promoting T24T cell invasion. Our previous results indicated that the overexpression of SOD2 in T24T cells contributes to the attenuation of cell migration, so its function in MMP-2 expression and cell invasion was further explored. As shown in Fig. 6F, MMP-2 was at the similar level across T24T nonsense and T24T shSOD2 cells, and the relative cell invasive abilities were also comparable between the T24T nonsense and T24T shSOD2 cells (Figs. 6G and 6H). These results together with above results of SOD2 regulation of cell migration, demonstrate that MMP-2 overexpression in T24T cells mediated their high invasion, while SOD2 is crucial for low migration abilities of T24T cells.

### Increased expression and nuclear translocation of nucleolin promotes mmp-2 mRNA stability in T24T cells

**Figure 7:**
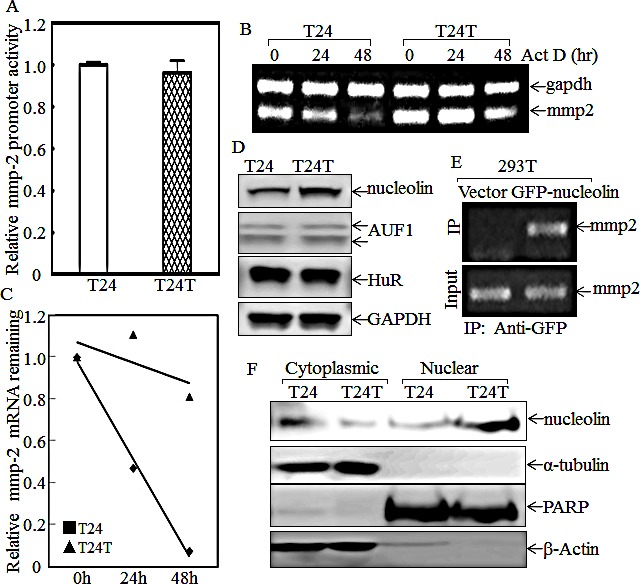
Nucleolin overexpression and nuclear translocation enhances mmp2 mRNA stability in T24T cells (A) The MMP-2 promoter transcription activity was evaluated by transfection of MMP-2 promoter-driven luciferase reporter. (B and C) Mmp-2 mRNA stabilities were evaluated by RT-PCR in presence of Act D in T24 and T24T (B) and the results were presented as mmp-2 mRNA remaining upon presence of Act D (C). (D) The cell extracts from T24 and T24T were subjected to Western Blot as indicated. (E) GFP-nucleolin was transfected into 293T cells and GFP-nucleolin protein was pulled down with anti-GFP beads. The mRNAs bound to nucleolin protein were used to carry out RT-PCR for determination of mmp-2 mRNA. (F) The cytoplasmic and nuclear protein extracted from T24 and T24T cells were subjected to Western Blot as indicated.

**Figure 8:**
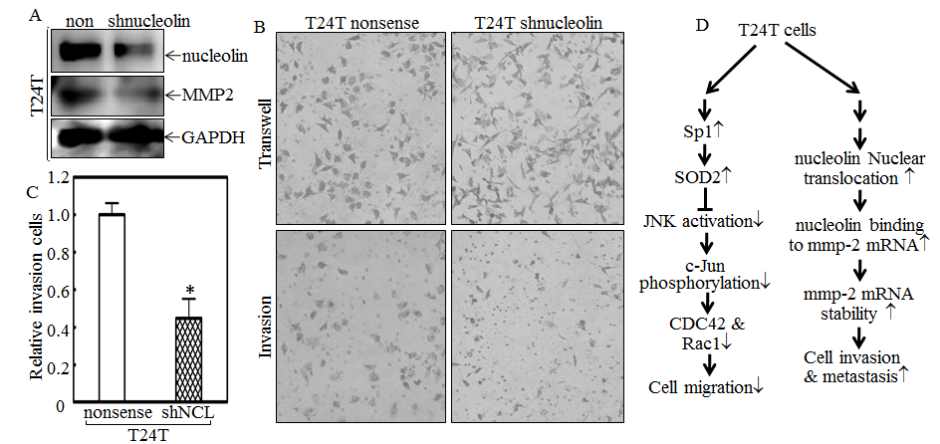
Nucleolin overexpression contributes to MMP-2 protein expression and cancer cell invasion of T24T cells (A and B) The cell extracts from T24T nonsense and T24T shNucleolin transfectants were subjected to Western blot as indicated (A), or invasion assays (B). The migration ability was determined using the empty insert membrane without the matrigel, while the invasion ability was evaluated by using the same system except that the matrigel was applied. (C) The invasion rate was normalized with the insert control according to the manufacturer's instruction and the results were presented as the invasion cells relative to T24T nonsense control transfectant. (D) The schematic of SOD2 regulation of human bladder T24T cell migration and invasion.

Because both MMP-2 protein and mRNA levels were higher in T24T cells, an MMP-2 promoter-driven luciferase reporter was transfected into T24 and T24T cells and the stable transfectants were used to compare the MMP-2 transcriptional activities between two cells. The results showed that MMP-2 promoter transcriptional activity in T24T cells was comparable to that in T24 cells (Fig.7A), indicating that the upregulation of MMP-2 in T24T cells didn't occur at the transcriptional level. With the possibility of transcriptional regulation out, we exploited mRNA stability regulation as a possible mechanism for the increased mmp-2 mRNA levels that we observed in T24T cells. After treatment with actinomycin D (Act D) for the indicated period time, RNA in T24 and T24T cells was exacted as described in “Materials and Methods”. The RT-PCR results indicated that the half-life of mmp-2 mRNA in T24T cells was much longer than in T24 cells (Fig.7B and 7C), suggesting that T24T has a high MMP-2 expression due to increased mmp-2 mRNA stability. AUF1, also known as AU-rich element RNA-binding protein 1, is a protein that can bind to RNA AU-rich sites and contribute to RNA degradation [33]. HuR is a protein that can also bind to AU-rich elements in RNA, but it instead increases the stability of the RNA [34]. Nucleolin is a protein, primarily in the nucleolus, that has four RNA-binding domains that can stabilize mRNA [35]. To determine the upstream pathway regulating mmp-2 mRNA stability, AUF1, HuR and nucleolin were tested in T24 and T24T cells. As the data shows in Fig.7D, the protein levels of both AUF1 and HuR in T24 and T24T were compared, but only nucleolin was elevated in T24T cells. The results obtained from the RNA-IP assay also showed that nucleolin can specific bind to mmp-2 mRNA (Fig.7E). Following this, we compared the distribution of nucleolin in paired T24 and T24T cells. The cytoplasmic and nuclear proteins of T24 and T24T cells were extracted, and as the data shows, the nuclear nucleolin was higher in T24T cells than in T24 cells (Fig. 7F). To further determine role of nucleolin in MMP-2 regulated T24T cell invasion, we constructed the stable nucleolin shRNA T24T transfectants (Fig.8A). The knockdown of nucleolin expression resulted in MMP-2 expression reduction (Fig. 8A), along with the impaired cell invasive abilities (Fig.8B and 8C). Thus, our results demonstrate that nucleolin enhances mmp-2 mRNA stability, increasing the invasiveness of T24T cells and the schematic regulation of human bladder T24T cell migration and invasion was shown in Fig. 8D.

## DISCUSSION

Bladder cancer invasion and metastasis is the leading factor that causes death [36]. Also, the factors that promote cancer cell migration usually enhance cancer cell invasion and metastasis [11, 37]. However, some recent studies indicate that cell migration is not consistent with the tendency of cell metastasis or invasion. The Wnt/β-Catenin signaling pathway is implicated in reducing melanocyte cell migration but promoting melanoma cell metastasis [38]. p120 catenin is required for HER2/ErbB2-induced breast cancer cell migration through the activation of CDC42 and Rac1 expression, while the overexpression of p120 in MCF-10A cells has no effect on cell invasion [39]. α-catenin is also implicated in inducing human colonic cancer cell migration but not cell invasion [40]. Soluble L1CAM, which is involved in promoting breast cancer cell migration, has no effect on regulating cell invasion [41]. To explore the migration and invasion activities of the bladder cancer T24/T24T cell system, the wound healing and cell invasion assays were performed and the relevant potential mechanisms were also elucidated in current studies. Our data demonstrated that cell migration related proteins CDC42 and Rac1 were suppressed in highly metastatic T24T cells, and their cell migration is inhibited in comparison to that of the less metastatic parental T24 cells. Our further studies revealed that highly metastatic T24T cells have increased MMP-2 expression, which upregulates T24T invasion, whereas overexpression of SOD2 mediated the inhibition of cell migration of T24T cells.

Oxidative stress has been reported to play the pivotal factor for inducing cancer development and metastasis [42-44], and SOD2, a critical component in regulation of reactive oxygen species [11], is also thought to be closely associated with bladder cancer invasion and metastasis. Recent studies have shown that SOD2 is consistently increased in high-grade and advanced-stage bladder cancer and is implicated in promoting cancer cell migration in tongue squamous cells [13, 19]. Our current studies showed that in T24T cells, although the expression of SOD2 was elevated, cell migration was attenuated compared to those in their parental T24 cells with low level of SOD2 expression. Also, suppressing SOD2 expression remarkably enhanced T24T cell migration. Those results demonstrate that SOD2 might function as an inhibitor for cell migration in paired T24/T24T bladder cancer cells. Importantly, we found that the increased SOD2 expression in T24T cells occurred at the transcriptional level, which was due to the enhanced Sp1 expression and transactivation in T24T cells. Moreover, the cell migration activity in T24T shSp1 cells was profoundly enhanced as compared with that of T24T nonsense cells. Our results demonstrated that transcriptional factor Sp1 overexpression and activation is initial factor in promoting SOD2 expression, which subsequently decreased T24T cell migration abilities.

Previous reports indicate that mitogen-activated protein kinase (MAPK) JNK is an enhancer of cell migration [29, 45]. In bladder cancer cells, MAPKs/AP-1 activation is mediated by interleukin-20 and also enhances cell migration inhuman bladder cancer T24 and U5637 [46]. SOD2 can also regulate MAPKs activation, and overexpression of SOD2 remarkably inhibits EGF-induced MAPKs activation in vascular smooth muscular cells [47]. We anticipated that SOD2 might regulate T24/T24T cell migration via the suppression of MAPK activation. This notion was greatly supported by the results obtained from current studies. In metastatic T24T cells, functional JNK was significantly reduced compared to the non-metastatic parental T24 cells. Knockdown of SOD2 resulted in a significant increase in JNK activation. Consistently, c-Jun phosphorylation and AP-1 activity was reduced in T24T cells, and the reduced c-Jun phosphorylation and AP-1 activity could be restored by the knockdown of SOD2 expression in T24T cells (Fig.3D). All results indicate that SOD2 has an inhibitory effect on JNK/AP-1 activation in bladder cancer T24Tcells.

The small Rho-GTPases, consisting of CDC42, Rac, and Rho, are crucial for cell migration [48, 49]. As important members of the Rho family GTPases, CDC42 and Rac1 also play essential roles in promoting JNK activation induced by various stimuli [50, 51]. MKK4/SEK is well-known upstream kinase of JNK [52], and mixed lineage kinase 3 (MLK3) regulates the activation of MKK4/SEK [53]. CDC42 and Rac1 are required for activating MLK3 and subsequently triggering MKK4/SEK-JNK signal pathway [53, 54]. However, in this study the results showed that JNK was also involved in SOD2-mediated the feedback regulation of CDC42 and Rac1 expression. Specifically inhibiting the activation of JNK/c-Jun axis resulted in the suppression of CDC42 and Rac1 expression in T24 shSOD2 cells. The transwell migration assay also demonstrated that TAM67 successfully reduced the cell migration of T24T shSOD2 cells. Although previous studies report that CDC42 and Rac1 can activate JNK and regulate cancer cell migration [55, 56], our results from current studies demonstrated that JNK could also regulate CDC42 and Rac1 expression and subsequently mediate T24 cell migration. In general, the feedback regulation of JNK on CDC42 and Rac1 expression was required for the repression of cell migration.

Cancer metastasis involves multiple steps. First is stromal invasion, then intravasion into the blood system or immune system until a cancer cell arrives at a new organ, and lastly extravasion into the distant tissue to form a secondary tumor [57]. Stromal invasion is a key step involving the destruction of the basement membrane to the linked tissue, and is very important for cancer cell invasion [58]. MMP-2 and MMP-9 are two major MMPs playing a central role in extracellular matrix digestion because they can hydrolyze type IV collagen, which is the main component of the extracellular matrix. It has been reported that MMP-2 and MMP-9 are overexpressed and implicated in promoting cancer cell invasion and metastasis in many cancers [59-65]. For example, MMP-2 overexpression is required for breast cancer brain metastasis development [9]. MMP-2 and MMP-9 expression levels are also used as biomarkers for prostate cancer malignant progression and therapeutic effects [66]. MMP-2 expression has been reported to be regulated by Erks [9, 67], which can increase mmp-2 mRNA stability upon irradiation [68]. Our current studies showed that MMP-2, but not MMP-9, was specifically increased in metastatic T24T cells, and knockdown MMP-2 impaired invasive activity of T24T cells. However, Erks phosphorylation was decreased in T24T cell in comparison to that in T24 cell, which was inconsistent with the tendency of MMP-2 expression, revealing that Erks are not involved in upregulation of MMP-2 expression in T24T cells. Our further mechanistic studies indicated that nucleolin overexpression and nuclear translocation, as well as its binds to mmp-2 mRNA are essential for stabilizing mmp-2 mRNA in T24T cells and in turn ultimately increasing the MMP-2 protein expression and invasion abilities of T24T cells. Thus, our studies identify a novel function of nucleolin in stabilizing mmp-2 mRNA and promoting human bladder T24T cell invasion.

Collectively, we found the inversely relation between cell migration abilities and invasiveness in human bladder cancer T24/T24T cells. We demonstrated that SOD2 was a critical factor in regulating bladder cancer cell migration in T24/T24T cells. SOD2 suppressed CDC42 and Rac1 expression and reduced cell migration through the inhibiting activation of JNK/c-Jun axis, whereas MMP-2 was a crucial for T24T cell invasion. Importantly, we revealed that nucleolin overexpression and translocation into the nucleus enhanced mmp-2 mRNA stability, thus promoting T24T cell invasion; while Sp1 overexpression and activation played an important role in SOD2 transcription and protein overexpression, as well as inhibition of cell migration inT24T cells.

## MATERIALS AND METHODS

### Cell Lines, Plasmids, and Antibodies

T24 and T24T cells [69] were kindly provided by Dr. Dan Theodorescu (University of Colorado Comprehensive Cancer Center, Denver, CO, USA). The plasmid containing luciferase reporter under control of human SOD2 gene promoter was a gift from Dr. Evert (University of Bonn, Bonn, Germany) [70]. The human MMP-2 promoter luciferase was gifted from Dr. Yi Sun (Michigan University, Michigan,) [71]. The plasmid of AP-1 luciferase reporter was purchased from Stratagene (Santa Clara, CA, USA). The construct containing the three consensus binding sites of Sp1, Sp1 reporter was previously described [72], the plasmid TAM67, a dominant negative mutant of c-Jun, was described in previously study [73]. The construct of short hairpin RNA specific to Sp1(shSp1), SOD2(shSOD2), MMP-2(shMMP-2), nucleolin(shnuclcolin), and their nonsense control construct were purchased from Open Biosystem (Pittsburg, PA, USA). Plasmids were prepared by the Plasmid Preparation/Extraction Maxi kit from QIAGEN (Valencia, CA, USA). The specific antibodies for MMP-2, SOD2, HuR, Nucleolin and Sp1 were purchased from Santa Cruz Biotechnology, Inc. (Santa Cruz, CA, USA). CDC42, Rac1, RhoA, c-Jun, phosphor-c-Jun at Ser63, phosphor-c-Jun at Ser73, JNK, phosphor-JNK at Thr183/Tyr185, Erk, phosphor-Erk at Thr202/Tyr204, HSP70 and GAPDH antibodies were purchased from Cell Signaling Technology (Beverly, MA, USA). Antibodies against β-Actin were bought from Sigma (St. Louis, MO, USA). Antibodies against AUF1were bought from Aviva (San Diego, CA, USA). The inhibitors of SP600125 and PD98059 were from Calbiochem (San Diego, CA, USA).

### Cell Transfection

Cell transfections were performed with PolyJet^TM^ DNA *in Vitro* Transfection Reagent (SignaGen Laboratories, Rockville, MD, USA) according to the manufacturer's instructions. For stable transfection, cell cultures were subjected to hygromycin B (200-400 μg/mL), G418 (500-1000 μg/mL) or puromycin (0.2-0.3μg/mL) and cells surviving from the antibiotics selection were pooled as stable mass transfectants.

### Western Blot Analysis

Whole cell extracts were prepared with the cell lysis buffer (10 mM Tris-HCl, pH 7.4, 1% SDS, and 1 mM Na_3_VO_4_) as described in our previous studies [74, 75]. 30 μg of proteins were resolved by SDS-PAGE, transferred to a membrane, probed with the indicated primary antibodies, and incubated with the AP-conjugated secondary antibody. Signals were detected by the enhanced chemifluorescence Western blotting system as described in a previous report [76]. The images were acquired by scanning with the phosphoimager (model Storm 860; Molecular Dynamics, Sunnyvale, CA, USA).

### Luciferase Promoter Reporter Assay

SOD2 promoter luciferase reporter, MMP-2 promoter luciferase reporter, Sp-1 luciferase reporter or AP-1 luciferase reporter and pRL-TK were each transiently transfected into T24 and T24T cells. Twenty-four hours later, luciferase activity was determined using the luciferase Assay System kit (Promega, Madison, WI, USA). The results were normalized by internal TK signal. All experiments were done in triplicates and the results expressed as mean ± standard error.

### RT-PCR

Total RNA was extracted using the TRIzol reagent (Invitrogen, Grand Island, NY, USA) as described in the manufacturer's instructions. Specific primers (Invitrogen, Grand Island, NY, USA) were used for PCR amplification. The primers used in this study were: *human sod2* (Forward: 5′-gca gtg tgc ggc acc agc ag -3′, Reverse: 5′-tcc ctt ggc caa cgc ctc ct -3′), *human rac1*(Forward: 5′-tgc aaa gtg gta tcc tga ggt gcg -3′, Reverse: 5′-gag gcc tcg ctg tgt gag cg -3′), *human cdc42* (Forward: 5′-acg tga aag aaa agt ggg tgc ct -3′, Reverse: 5′-tag cag cac aca cct gcg gc -3′), *human mmp-2*(Forward:5′-caa gtg gga caa gaa cca ga -3′, Reverse:5′-cca aag ttg atc atg atg tc -3′), *human mmp-9*(Forward:5′-ggg acg cag aca tcg tca c -3′, Reverse:5′-tcg tca tcg tcg aaa tgg c -3′) and *human gapdh*(Forward: 5′-gat gat ctt gag gct gtt gtc -3′, Reverse: 5′-cag ggc tgc ttt taa ctc tg -3′).

### RNA-IP assay

293T cells were cultured in 10-cm dishes. When cell confluence reached 70~80%, cells were transiently transfected with GFP-nucleolin and its GFP vector control. Twenty four hours after the transfection, the cells were extracted by using polysomelysis buffer (10 mM HEPES pH 7; 100 mM KCl; 5 mM MgCl2; 25 mM EDTA; 0.5% IGEPAL; 2 mM DTT; 50 units/ml RNase OUT; 50 units/ml Superase IN; 0.2 mg/ml heparin; and complete proteinase inhibitor). The cell lysates were centrifuged at 14,000× g for 10 min at 4°C. The anti-GFP agarose beads A/G (Purchased from Vector laboratories, Burlingame, CA, USA ) were added into the supernatant and rotated overnight at 4°C in NET2 buffer (50 mMTris–HCl, pH 7.4, 150 mM sodium chloride, 1 mM magnesium chloride, 0.05% IGEPAL, 50 U/mL RNase OUT, 50 U/mL Superase IN, 1 mM dithiothreitol, and 30 mM EDTA). The beads were washed three times, and resuspended in 100 μL NET2 and 100 μL SDS-TE (20 mM Tris-HCl, pH 7.5, 2 mM EDTA, and 2% sodium dodecyl sulfate) and then incubated at 55°C for 30 min, mixing occasionally. The RNAs in the buffer of the beads were extracted by phenol-chloroform-isoamyl alcohol and RT-PCR was performed to identify the mRNA presented in the immune-complex.

### Wound Healing Assay

Cells were seeded in 6-well plate, and wounds were made using sterile pipette tips when the cells reached 80~90% confluence. Cells were washed with serum-free PBS and then cultured in fresh medium for the time periods indicated. The photographs were taken at the times indicated until the wounds were healed in a group. The wound area was quantified using cell migration analysis software (Muscale LLC, Scottsdale, AZ, USA).

### Cell Invasion Assay

The invasion kit was purchased from BD falcon; the invasion assay was performed according to the manufacturer's instruction in normal cell culture serum. After incubation of assay transwell, the cells both on the inside and outside of the chamber were fixed with 3.7% formalin for 2 min, washed twice, then transferred to 100% methanol for 20min, washed twice again, then finally the cells were stained by Giemsa (1:20 diluted with PBS) at RT for 15 min in the dark. They were again washed twice, then the non-invaded cells were scraped off with a cotton swab (PBS wetted) 4 times. The photographs were taken with an Olympus DP71, and the number of the cells was calculated by the software “image J”.

### Statistic analysis

The Student's t-test was utilized to determine significant differences. The differences were considered to be significant at a P≤0.05.
